# The Burden of Rheumatoid Arthritis: Findings from the 2019 Global Burden of Diseases Study and Forecasts for 2030 by Bayesian Age-Period-Cohort Analysis

**DOI:** 10.3390/jcm12041291

**Published:** 2023-02-06

**Authors:** Yuanqing Cai, Jianan Zhang, Jialin Liang, Mofan Xiao, Guangyang Zhang, Zhaopu Jing, Leifeng Lv, Kai Nan, Xiaoqian Dang

**Affiliations:** 1 Department of Orthopaedics, The Second Affiliated Hospital of Xi’an Jiaotong University, Xi’an 710006, China; 2 Zonglian College, Xi’an Jiaotong University, Xi’an 710054, China; 3 Department of Gastroenterology, The First Affiliated Hospital of Xi’an Jiaotong University, Xi’an 710063, China; 4 Department of Osteonecrosis & Joint Reconstruction Surgery, Honghui Hospital, Xi’an Jiaotong University, Xi’an 710054, China

**Keywords:** rheumatoid arthritis, global burden of disease, incidence, prevalence, disability-adjusted life years

## Abstract

Background: Rheumatoid arthritis (RA) is a key health issue worldwide. Due to early identification and effective treatment strategies, the disease pattern of RA has also changed. However, the most comprehensive and up-to-date information about the burden of RA and its trends in subsequent years is lacking. Objective: this study aimed to report the global burden of RA by sex, age, region, and forecast for 2030. Method: Publicly available data from the Global Burden of Diseases, Injuries, and Risk Factors Study (GBD) 2019 were used in this study. The trends in the prevalence, incidence, and disability-adjusted life years (DALYs) of RA from 1990 to 2019 were reported. The global burden of RA in 2019 was reported by a sex, age, and sociodemographic index (SDI). Finally, the trends in the following years were predicted by Bayesian age-period-cohort (BAPC) models. Results: Globally, the age-standardized prevalence rate increased from 207.46 (95% UI:189.99 to 226.95) in 1990 to 224.25 (95% UI: 204.94 to 245.99) in 2019, with an estimated annual percent change (EAPC) of 0.37% (95% CI: 0.32 to 0.42). Regarding the incidence, the age-standardized incidence rate (ASR) increased from 12.21 (95% UI: 11.13 to 13.38) to 13 (95% UI: 11.83 to 14.27) per 100,000 people from 1990 to 2019, with an EAPC of 0.3% (95% CI: 11.83 to 14.27). The age-standardized DALY rate also increased from 39.12 (95% UI: 30.13 to 48.56) per 100,000 people in 1990 to 39.57 (95% UI: 30.51 to 49.53) in 2019, with an EAPC of 0.12% (95% CI: 0.08% to 0.17%). There was no significant association between the SDI and ASR when the SDI was lower than 0.7, while there was a positive association between the SDI and ASR when the SDI was higher than 0.7 The BAPC analysis showed that the ASR was estimated to be up to 18.23 in females and approximately 8.34 per 100,000 people in males by 2030. Conclusion: RA is still a key public health issue worldwide. The global burden of RA has increased over the past decades and will continue to increase in the coming years, and much more attention should be given to early diagnosis and treatment to reduce the burden of RA.

## 1. Introduction

Rheumatoid arthritis (RA) is a common autoimmune disease characterized by chronic inflammation of joint tissue [1,2]. Infection, genetics, and environmental factors are thought to play a role in the development of RA [3,4]. Approximately half of the patients exhibit swelling, pain, and deformation of small joints, particularly interphalangeal joints and wrist joints [5]. More advanced cases might exhibit joint deformation and functional limitations [4], and some important internal organs, such as the lungs, heart, kidneys, and digestive tract might also be involved, with a continuous and recurrent process, which is a key health issues worldwide and causes a tremendous psychological burden on patients and an economic burden on society [6,7].

Since RA has a high disability rate, it is important to determine the global picture, especially the burden of RA, such as prevalence, incidence, and disability-adjusted life years (DALYs). Previous studies have reported the global burden of RA at the regional or national level based on WHO databases or old versions of the Global Burden of Diseases Study [8,9,10]. Cross et al. [11] first reported the global burden of RA in 2014 based on the GBD 2010 and drew attention to the global burden of RA. Sebbag et al. [9] reported the burden of RA as a panel of musculoskeletal diseases based on the GBD 2015 but did not focus on RA, and Safiri et al. [10] reported the newest global burden of RA based on the 2017 GBD but did not update it after that. Moreover, none of the previous studies predicted the trends in RA incidence in the following years, which would be helpful for policy-makers to assess the burden of RA in the near future and develop some strategies to reduce this burden. Thus, in the present work, we aimed to provide the most comprehensive and up-to-date information on the global burden of RA based on the GBD 2019 and predict its trends for 2030.

## 2. Materials and Methods

### 2.1. Overview and Data Sources

The Burden of Diseases, Injuries, and Risk Factors Study 2019 (GBD 2019) was published by the Institute for Health Metrics and Evaluation (IHME) [12], and all data were open source and available to all. It is the most comprehensive and detailed study of diseases, injuries, and risk factors worldwide.

A total of 204 countries and territories were covered by the GBD 2019, which estimated the burden of 369 diseases and injuries and 87 risk factors. A general overview of the methodology of the GBD 2019 and improvements from the previous version of GBD have been provided in previous publications [12,13]. Detailed information about fatal and nonfatal estimates used in the GBD 2019 can be found at (https://vizhub.healthdata.org/gbd-compare/india, accessed on 25 August 2022) and (http://ghdx.healthdata.org/gbd-results-tool, accessed on 25 August 2022).

### 2.2. Global, Regional, and Country-Specific Burden of RA

The IHME conducted a systematic review of the prevalence and incidence of RA in the population from 1980 to 2009 for the GBD 2010 and updated it for the GBD 2017. In the GBD 2019, the prevalence and incidence of RA were updated, providing the comprehensive and latest information about the burden of RA.

Depending on their sociodemographic index (SDI), countries were categorized into five classes: low, low–middle, middle, middle–high, and high. Geographically, the world was divided into 21 regions. According to the Global Health Data Exchange (GHDx) query tool (http://ghdx.healthdata.org/gbd-results-tool, accessed on 25 August 2022), RA prevalence, incidence, and DALYs from 1990 to 2019 were collected and reported by sex, country, and region.

### 2.3. Sociodemographic Index (SDI) and Risk Factors

The sociodemographic index (SDI) was used in this study to examine the association between socioeconomic status and age-standardized incidence rate (ASR). The SDI measures the overall economic development of a society based on educational attainment, per capita income level, and fertility rate; the SDI ranges from 0 to 1, with higher values representing higher levels of socioeconomic development [14]. Moreover, we obtained and reported the percentage of DALYs due to RA that were attributed to all risk factors reported by the GBD 2019 [13].

### 2.4. Statistical Analysis

Data on RA incidence, prevalence, and DALYs were described by year. The ASR, age-standardized rate of prevalence, and DALYs for RA were reported to reflect changes in RA incidence. The estimated annual percent change (EAPC) was defined as 100× (exp(β)–1); the 95% confidence interval (CI) of the EAPC was also determined by the fitted model [15,16]. Comparisons of 2019 data with 1990 data were used to determine the changes in each outcome. Furthermore, we also explored the association between the EAPC and the ASR and human development index (HDI).

Finally, we ran a Bayesian age-period-cohort (BAPC) analysis in R using the BAPC and INLA packages to predict the ASR from 2019 to 2030 by sex [17]. Age-period-cohort (APC) analysis was mainly used to analyze the changing trend in chronic disease incidence and mortality and to predict the change in disease burden in the future. Its considered factors include age, period, and cohort, but there is a linear relationship among the three factors, which may lead to the occurrence of nonunique parameter estimates. However, the Bayesian APC (BAPC) uses both sample information and prior information to obtain unique parameter estimates, and the obtained results are robust and reliable [18,19]. All data analyses were conducted using the open-source software R (version 4.2.1; R Foundation for Statistical Computing, Vienna, Austria).

## 3. Results

### 3.1. Global Level

The trends in the global burden of RA from 1990 to 2019 are summarized in Supplementary Table S1. As Supplementary Table S1 shows, globally, the age-standardized prevalence rate increased from 207.46 (95% UI: 189.99 to 226.95) in 1990 to 224.25 (95% UI: 204.94 to 245.99) in 2019, with an EAPC of 0.37%. Regarding the incidence, the ASR increased from 1990 to 2019, with an EAPC of 0.3%. The age-standardized DALY rate also increased from 39.12 (95% UI: 30.13 to 48.56) per 100,000 in 1990 to 39.57 (95% UI: 30.51 to 49.53) per 100,000 people in 2019, with an EAPC of 0.12%. The largest EAPC in the ASR, age-standardized rate of prevalence, and DALYs were observed in the Andean Latin America region, standardized. For SDI regions, the burden of RA in most SDI regions increased from 1990 to 2019, but interestingly, the age-standardized DALY rate in high SDI regions decreased from 47.62 (95% UI: 36.29 to 59.68) in 1990 to 45.02 (95% UI: 33.28 to 57.74) per 100,000 people in 2019.

**Figure 1:**
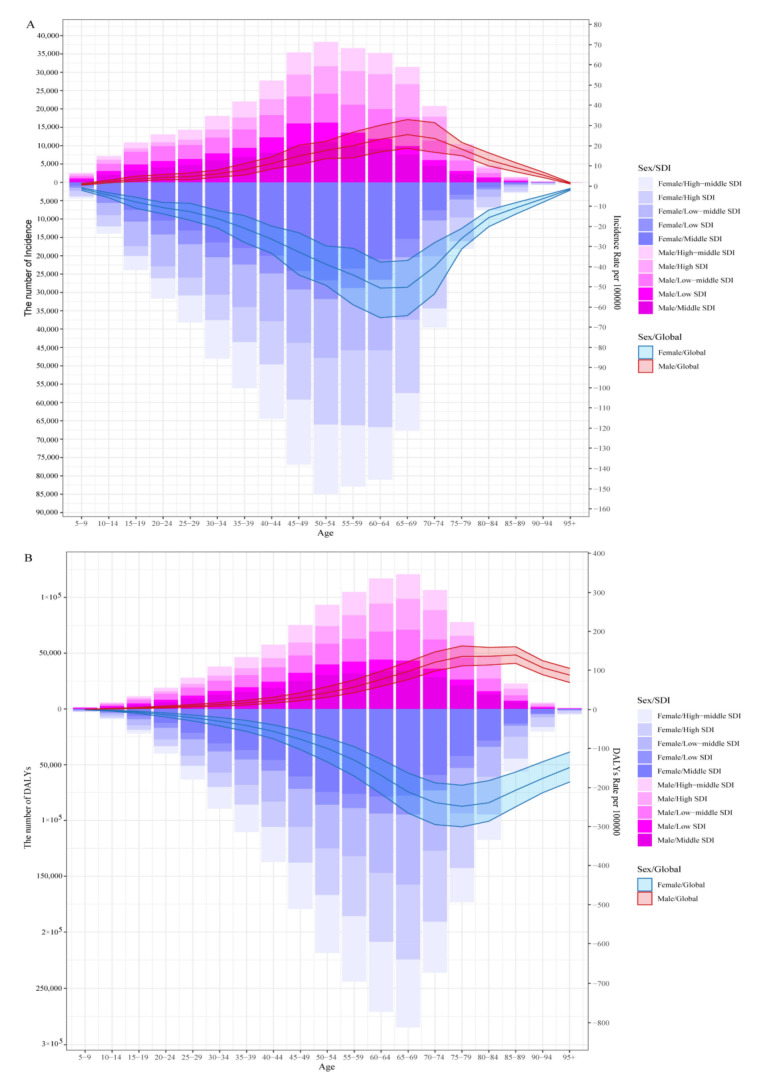
The number and age-standardized rates of incidence (**A**) and DALYs (**B**) by sex, age, and SDI in 2019. The incidence increased from 5 to 54 years and decreased from 55 years. The 50–54 age group had the highest incidence (**A**); the number of DALYs increased from 5 to 69 years and then decreased, and the 65–59 age group had the highest number of DALYs (**B**). SDI: sociodemographic index; DALYs: disability-adjusted life years.

The burden of RA in 2019 is summarized in Figure 1. As Figure 1A shows, the incidence increased among people aged 5 to 54 years and decreased among people aged 55 years and above. The 50–54 age group had the highest incidence; for males in the middle SDI region, the incidence was approximately 10,917, and for females in the middle SDI region, the incidence was approximately 26,685. Globally, the ASR among males increased from 5 to 69 years and decreased from 70. The highest ASR was found in the 65–69 age group, approximately 25.46 (95% UI: 18.66 to 32.93) per 100,000 people, while the ASR among females increased from 5 to 64 years and decreased from 65 years. The 60–64 age group had the highest ASR, approximately 50.55 (95% UI: 37.69 to 65.26) per 100,000 people. The DALYs of RA in 2019 are summarized in Figure 1B. The number of DALYs increased from 5 to 69 years and then decreased. The 65 to 59-year-old age group had the highest number of DALYs; specifically, the number was approximately 34,198.99 for males in the middle SDI region and approximately 77,713.24 for females.

### 3.2. National Level

**Figure 2:**
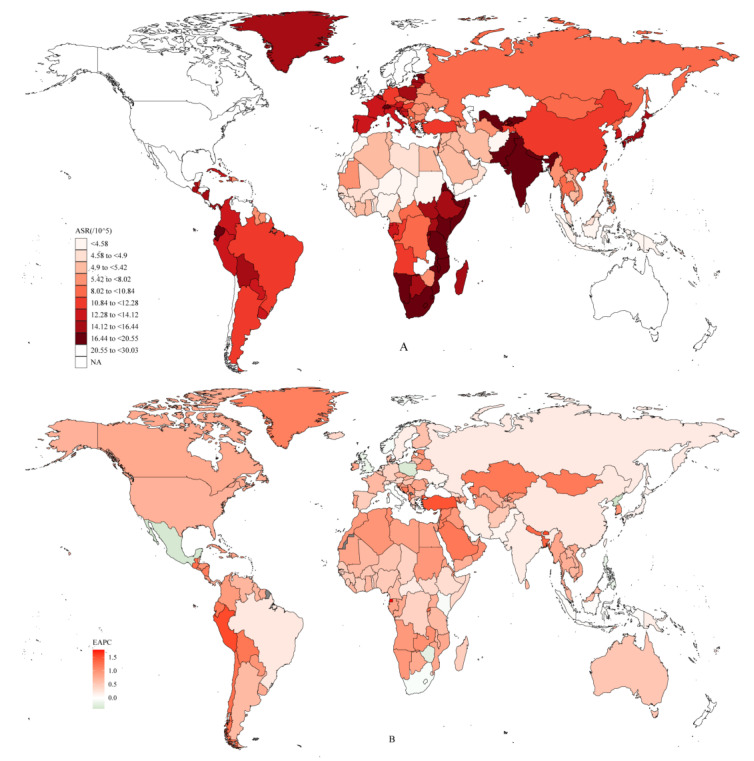
The global disease burden of rheumatoid arthritis for both sexes in 204 countries and territories. (**A**) The ASR of rheumatoid arthritis in 2019, the largest ASR was observed in Ireland, followed by Finland, Kazakhstan, Mexico, and Honduras. (**B**) The EAPC of rheumatoid arthritis in 2019: Equatorial Guinea had the highest increase rate of ASR, followed by Bhutan, Peru, Turkey, and Bangladesh. ASR: age-standardized incidence rate; EAPC: estimated annual percent change.

The global burden of RA at the national level is summarized in Figure 2. The ASR varied from 3.47 (95% UI: 2.96 to 4.1) to 30.03 (95% UI: 26.97 to 33.31) per 100,000 people (Figure 2A). The largest ASR was observed in Ireland (30.03; 95% UI: 26.97 to 33.31 per 100,000 people), followed by Finland (27.89; 95% UI: 25.5 to 30.76 per 100,000), Kazakhstan (25.5; 95% UI: 23.45 to 28.01 per 100,000), Mexico (25.43; 95% UI: 22.94 to 28.03 per 100,000 people), and Honduras (25.06; 95% UI: 22.49 to 27.68 per 100,000 people). To obtain a picture of the trends in ASR from 1990 to 2019, the EAPCs were calculated and summarized in Figure 2B. As Figure 2B shows, the ASR increased in most countries. Equatorial Guinea had the highest increase rate of ASR (EAPC: 1.78%; 95% CI: 1.60% to 1.96%), followed by Bhutan (EAPC: 1.54%; 95% CI: 1.47% to 1.60%), Peru (1.53%; 95% CI: 1.47% to 1.59%), Turkey (EAPC: 1.47%; 95% CI: 1.38% to 1.56%), and Bangladesh (EAPC: 1.36%; 95% CI: 1.27% to 1.45%), while in some countries, the ASR decreased from 1990 to 2019, such as in Italy (EAPC: −0.035%; 95% CI: −0.041% to −0.029%), Kenya (EAPC: −0.062%; 95% CI: −0.073% to −0.051%), and in the United Kingdom (EAPC: −0.14%; 95% CI: −0.22% to −0.067%).

### 3.3. The Association between the ASR and SDI, the EAPC and ASR, and HDI

**Figure 3:**
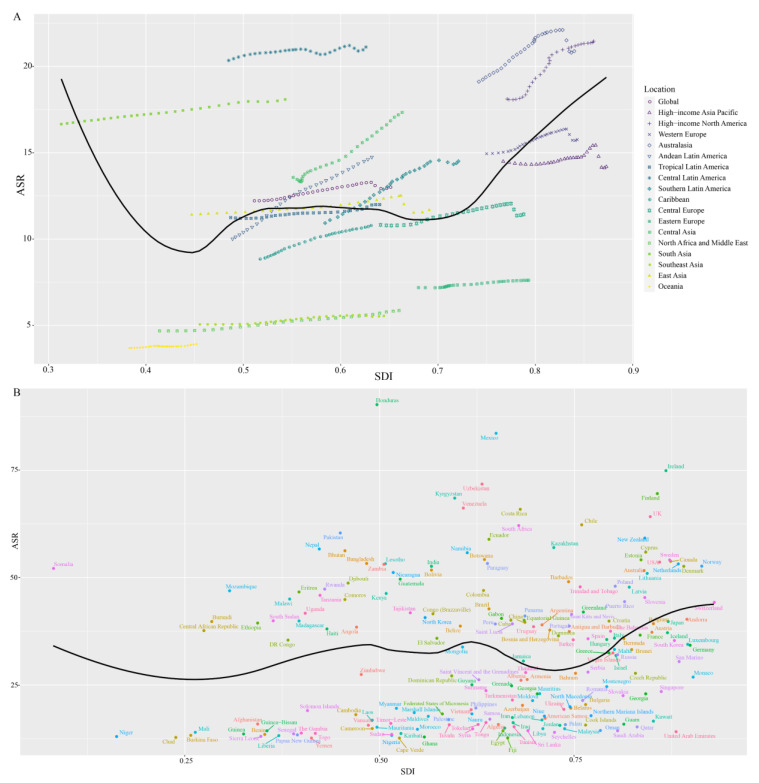
The association between the ASR and SDI at the regional (**A**) and national (**B**) levels. There was no significant association between the SDI and ASR when the SDI was lower than 0.7, while there was a positive association between the SDI and ASR when the SDI was higher than 0.7. ASR: age-standardized incidence rate; SDI: sociodemographic index.

We further explored the correlation between the SDI and ASR at the regional and national levels and summarized the results in Figure 3. As Figure 3A shows, there was no significant association between the SDI and ASR when the SDI was lower than 0.7, while there was a positive association between the SDI and ASR when the SDI was higher than 0.7. In some regions, such as the high-income Asian Pacific, Australian, Central Latin American, and Central Asian regions, the ASR levels were higher than predicted. This correlation was also confirmed at the national level when the SDI was larger than 0.7 (Figure 3B), indicating that in those regions and countries with a higher SDI, the ASR of RA might be higher. Moreover, since the EAPC varied among countries, we also tried to determine EAPC-related factors, especially for the ASR and HDI. The results are summarized in Supplementary Figure S1, and neither of these two factors was associated with the EAPC of RA.

### 3.4. Risk Factors

**Figure 4:**
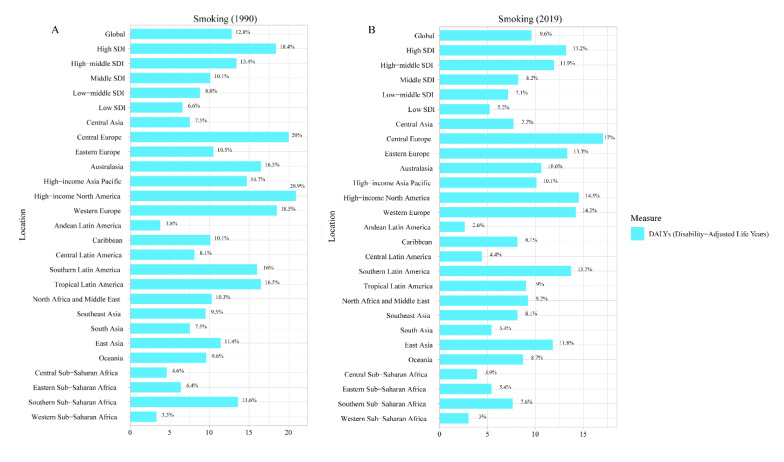
Risk factors for rheumatoid arthritis in 1990 (**A**) and 2019 (**B**). The RA-related risk factor analysis results showed that smoking was the only risk factor for DALYs due to RA based on the 2019 GBD.

The RA-related risk factor analysis results showed that smoking was the only risk factor for DALYs due to RA based on the 2019 GBD. In 1990, globally, 12.8% of DALYs were attributed to RA due to smoking (Figure 4A). Interestingly, this percentage decreased to 9.6% by 2019. This phenomenon was also found in SDI regions, from 18.4%, 13.4%, 10.1%, 8.8%, and 6.6% in high SDI, high–middle SDI, middle SDI, low–middle SDI, and low SDI regions, respectively, in 1990, but 13.2%, 11.9%, 8.2%, 7.1% and 2.5% in high SDI, high–middle SDI, middle SDI, low–middle SDI and low SDI regions (Figure 4B), respectively, in 2019.

### 3.5. ASR Forecasts for 2030 by Bayesian Age-Period-Cohort Analysis

**Figure 5:**
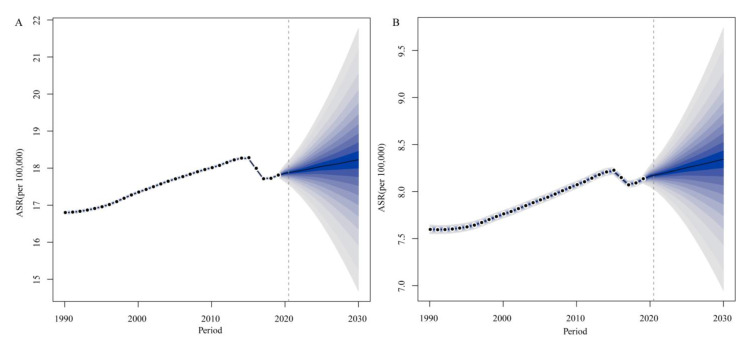
Trends in ASR from 2019 to 2030 among females (**A**) and males (**B**) predicted by Bayesian age-period-cohort (BAPC) models. ASR: age-standardized incidence rate. Generally, the ASR will increase in the following years both among males and females.

The BAPC analysis results are summarized in Figure 5. Generally, the ASR will increase in the following years. As Figure 5A shows, the ASR will continue to increase at a moderate rate among females and is estimated to be up to 18.23 per 100,000 people by 2030. Figure 5B shows that the trends in the ASR among males were similar to those among females, and the ASR is estimated to be approximately 8.34 per 100,000 people in 2030. This result indicates that the global burden of RA will increase and might be larger than predicted since the extension of lifespan.

## 4. Discussion

RA is a key public health issue worldwide and can progress to joint deformities and disabilities over time, along with internal complications, such as rheumatoid heart disease [20,21,22]. RA is a difficult medical issue to treat due to its recurrent course, high disability rate, high health care costs, and loss of labor. As lifestyles, dietary habits, and global living conditions have changed in recent decades, the disease pattern of RA has changed greatly [23,24]; therefore, it is important to obtain the newest picture of RA globally to provide basic information for policy-makers to implement measures. To the best of our knowledge, the present work showed the most comprehensive and up-to-date overview of the global burden of RA.

The present study showed that the global burden of RA increased between 1990 and 2019, and this increase might be attributed to the growth of the population and the improvement of diagnostic methods. Of course, more data and cases were included in subsequent GBD updates. However, compared with the newest report published by Safiri et al. [10] based on the GBD 2017, interestingly, the trend in the global burden of RA decreased slightly. In the GBD 2017, the RA prevalent cases were reduced from 2017 to 2019 (from 19,965,115 to 18,583,481) and the number of incident cases was 1,204,599 (with an ASR of 14.9 per 100,000 people) in GBD 2017, which decreased to 1,074,391 (with an ASR of 13 per 100,000 people) in GBD 2019. The number of DALYs attributed to RA was 3,492,036 with an age-standardized rate of 43.3 per 100,000 people in the GBD 2017, which decreased to approximately 3,262,589 with an age-standardized rate of 39.57 per 100,000 people in GBD 2019. However, studies performed by Cross et al. [8] did not include a comprehensive analysis of the global burden of RA, limiting further comparison with the present work. This comparison showed that the trends in the global burden of RA increased from 1990 to 2019 but decreased slightly from 2017 to 2019, indicating that many instructive measures have been taken in recent years, which should be continued in the following years.

Similarly, Cross et al. [8], Safiri et al. [10], and the present work all showed that the global burden of RA was larger among females than males, which might be attributed to sex hormones. Generally, as anti-inflammatory hormones, androgens suppress both humoral and cellular immune responses, whereas estrogens enhance humoral immune responses. A study of rheumatoid arthritis patients found that their bodily fluids (blood, synovial fluid, smears, saliva) were low in both gonadal and adrenal androgen levels (testosterone, dihydrotestosterone, dehydroepiandrosterone sulfate) [25], suggesting that decreased levels of immune-suppressive androgens might be detrimental. Thus, much more attention should be given to females with regard to how to prevent and treat RA effectively; moreover, the therapeutic modulation of sex hormone balance should be taken into consideration for advanced biological treatments for RA due to its crucial factor in the regulation of immune and inflammatory responses.

In contrast to previous studies, the present study also tried to predict the ASR for 2030 among females and males by BAPC analysis for the first time, and we showed that the trends in the ASR among females and males will keep increasing at a moderate rate from 2019 to 2030, which was estimated to be 18.23 and 8.34 per 100,000 people for females and males, respectively. Thus, reducing the global burden of RA is an urgent issue worldwide. Based on the risk factors analyzed, smoking was the only risk factor for RA, which has also been reported by Safiri et al. [10]. To reduce the global burden of RA, any modifiable risk factors should be reduced, so quitting smoking is highly recommended. Moreover, a more sensitive diagnostic method needs to be established [26]. Since early diagnosis is very important for reducing the burden of RA, it has been reported that most of the joint destruction caused by RA occurs within two years of disease onset [27], and the progress of the disease could be delayed if effective and timely treatment is provided during this time period. Furthermore, early treatment, such as nonsteroidal anti-inflammatory drugs and immunosuppressive drug therapy, should be initiated [27,28]. In addition, regular follow-ups are imperative for RA patients to ensure that the disease is well controlled, preventing joint deformities or even disability [28,29].

The present work, which was based on the 2019 GBD, provides the most comprehensive and up-to-date information about the global burden of RA but still has some limitations: (1) RA included in the 2019 GBD did not present different stages of RA, so it is impossible to analyze the burden of RA based on disease courses; (2) the 2019 GBD included fewer RA related risk factors; thus, we could not identify risk factors for individual regions and countries, which would be helpful for policy-makers to take more targeted measures to reduce the burden of RA. It was also impossible to investigate to what extent the increased global burden can be attributed to population growth.

## 5. Conclusions

In conclusion, RA is a key health issue worldwide. The global burden of RA has increased over the past decades and will continue to increase in the following years, and much more attention should be given to the early diagnosis and treatment of RA worldwide.

## Data Availability

All data generated during this study are available at (https://vizhub.healthdata.org/gbd-results/result/ea5cbddf88ae18d519f84ccc6d1fb879, accessed on 25 August 2022) and Supplementary Materials. Further information is available from the corresponding author.

## Supplementary Materials

The following supporting information can be downloaded at: https://www.mdpi.com/article/10.3390/jcm12041291/s1, Table S1: The burden of rheumatoid arthritis in 1990 and 2019 for both sexes and all locations, with EAPC from 1990 and 2019; Figure S1: The association between EAPC and ASR analysis results: there was no significant association between EAPC and ASR.
