# Supplementary material for: The Burden of Rheumatoid Arthritis: Findings from the 2019 Global Burden of Diseases Study and Forecasts for 2030 by Bayesian Age-Period-Cohort Analysis
Source: J Clin Med. 2023 Feb 6;12(4):1291. doi: 10.3390/jcm12041291 (PMC9959633; doi:10.3390/jcm12041291)
Supplement: Supplementary file 1 [file jcm-12-01291-s001.zip › jcm-2157240-supplementary.pdf]

# Suppementary Materials

**Table S1.** The burden of rheumatoid arthritis in 1990 and 2019 for both sexes and all locations, with EAPC from 1990 and 2019.

| | Prevalence | | | Incidence | | | DALYs | | |
|---|---|---|---|---|---|---|---|---|---|
| **Location** | **Age-Standardized Esti-mates (1990; 95%UI)** | **Age-Standardized Estimates (2019; 95%UI)** | **EAPC (95%CI)** | **Age-Standardized Estimates (1990; 95%UI)** | **Age-Standardized Estimates (2019; 95%UI)** | **EAPC (95%CI)** | **Age-Standardized Estimates (1990; 95%UI)** | **Age-Standardized Estimates (2019; 95%UI)** | **EAPC (95%CI)** |
| **Global** | 207.46 (189.99 to 226.95) | 224.25 (204.94 to 245.99) | 0.37% (0.32 to 0.42) | 12.21 (11.13 to 13.38) | 13 (11.83 to 14.27) | 0.3% (0.26 to 0.34) | 39.12 (30.13 to 48.56) | 39.57 (30.51 to 49.53) | 0.12% (0.08 to 0.17) |
| **High SDI** | 264.73 (245.29 to 286.87) | 286.7 (265.15 to 311.79) | 0.41% (0.35 to 0.47) | 15.95 (14.73 to 17.25) | 17.13 (15.77 to 18.61) | 0.38% (0.32 to 0.45) | 47.62 (36.29 to 59.68) | 45.02 (33.28 to 57.74) | -0.12% (-0.15 to -0.09) |
| **High-middle SDI** | 188.01 (171.49 to 206.38) | 208.51 (190.26 to 228.62) | 0.48% (0.42 to 0.54) | 10.17 (9.26 to 11.23) | 11.24 (10.24 to 12.38) | 0.45% (0.4 to 0.51) | 34.02 (25.74 to 42.78) | 35.01 (26.33 to 44.52) | 0.18% (0.13 to 0.24) |
| **Middle SDI** | 187.29 (169.79 to 206.45) | 205.83 (186.86 to 227.03) | 0.41% (0.36 to 0.47) | 10.65 (9.63 to 11.76) | 11.48 (10.41 to 12.69) | 0.31% (0.27 to 0.35) | 35.69 (27.56 to 44.59) | 37.61 (29.11 to 47.14) | 0.31% (0.22 to 0.39) |
| **Low-middle SDI** | 206.1 (187.42 to 226.87) | 236.03 (214.92 to 259.95) | 0.51% (0.48 to 0.54) | 13.89 (12.63 to 15.29) | 15.31 (13.9 to 16.84) | 0.36% (0.34 to 0.38) | 43.47 (34.07 to 53.54) | 48.38 (37.95 to 59.54) | 0.41% (0.38 to 0.44) |
| **Low SDI** | 176.28 (159.36 to 194.73) | 197.96 (178.69 to 218.3) | 0.48% (0.44 to 0.51) | 12.01 (10.85 to 13.21) | 13.27 (12.06 to 14.56) | 0.41% (0.39 to 0.44) | 35.2 (27.49 to 44.42) | 38.07 (29.29 to 47.88) | 0.29% (0.26 to 0.32) |
| **Andean Latin America** | 177.78 (155.08 to 204.78) | 270.38 (236.5 to 309.49) | 1.49% (1.42 to 1.56) | 10.01 (8.93 to 11.31) | 14.74 (13.16 to 16.62) | 1.39% (1.32 to 1.45) | 35.29 (27.27 to 44.28) | 46.17 (34.27 to 59.1) | 0.94% (0.83 to 1.04) |
| **Australasia** | 299.72 (268.16 to 338.41) | 331.81 (294.4 to 374.68) | 0.46% (0.36 to 0.56) | 19.12 (17.25 to 21.24) | 20.89 (18.75 to 23.34) | 0.43% (0.32 to 0.55) | 56.49 (42.91 to 71.42) | 52.75 (38.71 to 68.08) | -0.15% (-0.19 to -0.11) |
| **Caribbean** | 173.66 (150.61 to 199.98) | 216.7 (189.25 to 248.47) | 0.81% (0.76 to 0.86) | 8.84 (7.78 to 10.03) | 10.77 (9.55 to 12.17) | 0.72% (0.67 to 0.77) | 31.67 (23.54 to 40.11) | 36.11 (26.56 to 46.29) | 0.5% (0.48 to 0.51) |
| **Central Asia** | 243.88 (221.58 to 271.56) | 318.15 (289.66 to 352.63) | 1.07% (0.97 to 1.17) | 13.57 (12.47 to 14.89) | 17.33 (15.91 to 18.97) | 1% (0.9 to 1.09) | 41.29 (30.03 to 52.86) | 51.16 (37.6 to 65.3) | 0.69% (0.61 to 0.77) |
| **Central Europe** | 198.17 (180.69 to 218.5) | 211.46 (190.67 to 234.93) | 0.36% (0.3 to 0.42) | 10.82 (9.73 to 11.99) | 11.44 (10.2 to 12.72) | 0.35% (0.26 to 0.44) | 36.09 (26.96 to 45.67) | 34.08 (25.02 to 44.13) | -0.11% (-0.18 to -0.03) |
| **Central Latin America** | 346.98 (318.59 to 379.44) | 366.08 (335.61 to 399.02) | 0.13% (0.1 to 0.16) | 20.35 (18.54 to 22.23) | 21.12 (19.29 to 23.07) | 0.08% (0.05 to 0.11) | 72.37 (56.77 to 90.73) | 68.69 (52.71 to 86.7) | -0.17% (-0.22 to -0.12) |
| **Central Sub-Saharan Africa** | 152.48 (134.29 to 171.99) | 175.89 (155.72 to 198.71) | 0.57% (0.52 to 0.62) | 9.18 (8.12 to 10.32) | 10.57 (9.39 to 11.92) | 0.57% (0.51 to 0.62) | 37.13 (27.5 to 47.02) | 36.53 (26.88 to 47.19) | -0.11% (-0.17 to -0.06) |
| **East Asia** | 205.48 (187.04 to 227.01) | 215.89 (196.34 to 238.29) | 0.34% (0.23 to 0.46) | 11.41 (10.31 to 12.65) | 11.68 (10.56 to 12.89) | 0.21% (0.12 to 0.3) | 38.92 (29.85 to 49.62) | 39.3 (30.11 to 49.71) | 0.31% (0.14 to 0.48) |
| **Eastern Europe** | 150.7 (136.89 to 165.49) | 159.93 (144.81 to 175.61) | 0.26% (0.24 to 0.28) | 7.19 (6.45 to 8) | 7.61 (6.83 to 8.47) | 0.24% (0.23 to 0.25) | 28.97 (22.03 to 36.32) | 29 (22.08 to 36.95) | -0.09% (-0.15 to -0.04) |
| **Eastern Sub-Saharan Africa** | 228 (205.51 to 253.42) | 259.36 (233.8 to 288.04) | 0.55% (0.51 to 0.59) | 15.05 (13.6 to 16.61) | 17.02 (15.4 to 18.77) | 0.52% (0.48 to 0.56) | 42.38 (31.27 to 56.17) | 43.79 (32.22 to 57.93) | 0.14% (0.11 to 0.16) |
| **High-income Asia Pacific** | 234.31 (211.78 to 258.3) | 240.81 (218.19 to 267.22) | 0.28% (0.19 to 0.36) | 14.5 (13.05 to 15.99) | 14.19 (12.75 to 15.65) | 0.11% (0.02 to 0.2) | 46.61 (35.04 to 58.57) | 38.31 (28.14 to 49.8) | -0.63% (-0.71 to -0.56) |
| **High-income North America** | 290.68 (276.67 to 307.14) | 343.38 (324.49 to 364.88) | 0.73% (0.67 to 0.79) | 18.12 (16.98 to 19.4) | 21.46 (20.02 to 23.09) | 0.72% (0.66 to 0.79) | 47.73 (35.95 to 60.34) | 53.53 (39.82 to 68.25) | 0.47% (0.38 to 0.56) |
| **North Africa and Middle East** | 94.02 (83.07 to 106.69) | 120.59 (107 to 135.7) | 0.94% (0.91 to 0.97) | 4.68 (4.15 to 5.3) | 5.86 (5.18 to 6.63) | 0.85% (0.82 to 0.88) | 16.05 (11.65 to 20.91) | 19.04 (13.91 to 24.87) | 0.66% (0.62 to 0.69) |
| **Oceania** | 76.11 (64.74 to 89.32) | 81.17 (69.34 to 95.33) | 0.14% (0.11 to 0.18) | 3.69 (3.2 to 4.26) | 3.92 (3.4 to 4.51) | 0.13% (0.1 to 0.17) | 12.36 (8.8 to 16.43) | 13.77 (9.89 to 18.4) | 0.37% (0.34 to 0.39) |
| **South Asia** | 230.54 (209.14 to 252.67) | 258.01 (234.42 to 283.88) | 0.4% (0.39 to 0.42) | 16.66 (15.08 to 18.36) | 18.09 (16.35 to 19.94) | 0.29% (0.28 to 0.3) | 49.99 (39.16 to 61.89) | 53.36 (41.81 to 65.99) | 0.18% (0.14 to 0.21) |

| | | | | | | | | | |
|---|---|---|---|---|---|---|---|---|---|
| **Southeast Asia** | 94.03<br>(83.47 to 106.06) | 106.36<br>(94.45 to 120.02) | 0.52%<br>(0.49 to 0.56) | 5.05<br>(4.47 to 5.68) | 5.54<br>(4.92 to 6.24) | 0.42%<br>(0.38 to 0.46) | 16.17<br>(11.99 to 20.98) | 18.48<br>(13.76 to 23.64) | 0.54%<br>(0.5 to 0.58) |
| **Southern Latin America** | 195.56<br>(173.16 to 222.19) | 263.56<br>(238.3 to 295.09) | 1.03%<br>(0.95 to 1.11) | 10.93<br>(9.78 to 12.24) | 14.51<br>(13.16 to 16.09) | 1.01%<br>(0.92 to 1.1) | 37.35<br>(27.96 to 47.1) | 45.77<br>(34.05 to 58.57) | 0.78%<br>(0.73 to 0.84) |
| **Southern Sub-Saharan Africa** | 327.49<br>(297.7 to 362.02) | 320.35<br>(291.15 to 354.45) | 0.05%<br>(-0.01 to 0.11) | 17.51<br>(15.69 to 19.48) | 17.16<br>(15.35 to 19.11) | 0.03%<br>(-0.01 to 0.08) | 66.91<br>(51.84 to 82.86) | 56.76<br>(43.29 to 71.95) | -0.75%<br>(-0.97 to -0.54) |
| **Tropical Latin America** | 249.2<br>(224.56 to 274.85) | 272.89<br>(245.92 to 301.45) | 0.31%<br>(0.29 to 0.33) | 11.25<br>(10.07 to 12.59) | 12<br>(10.74 to 13.41) | 0.23%<br>(0.2 to 0.25) | 40.03<br>(29.04 to 51.63) | 42.82<br>(31.44 to 55.05) | 0.28%<br>(0.26 to 0.3) |
| **Western Europe** | 256.73<br>(232.01 to 283.45) | 269.44<br>(242.5 to 299.68) | 0.24%<br>(0.19 to 0.29) | 14.94<br>(13.64 to 16.35) | 15.76<br>(14.35 to 17.32) | 0.28%<br>(0.22 to 0.35) | 46.37<br>(35.1 to 58.69) | 42.22<br>(30.83 to 54.67) | -0.27%<br>(-0.3 to -0.24) |
| **Western Sub-Saharan Africa** | 57.35<br>(50.45 to 65.21) | 70.3<br>(61.89 to 79.98) | 0.63%<br>(0.58 to 0.69) | 3.9<br>(3.45 to 4.4) | 4.63<br>(4.09 to 5.23) | 0.54%<br>(0.49 to 0.59) | 10.56<br>(7.97 to 13.59) | 13.29<br>(9.8 to 17.09) | 0.8%<br>(0.77 to 0.83) |

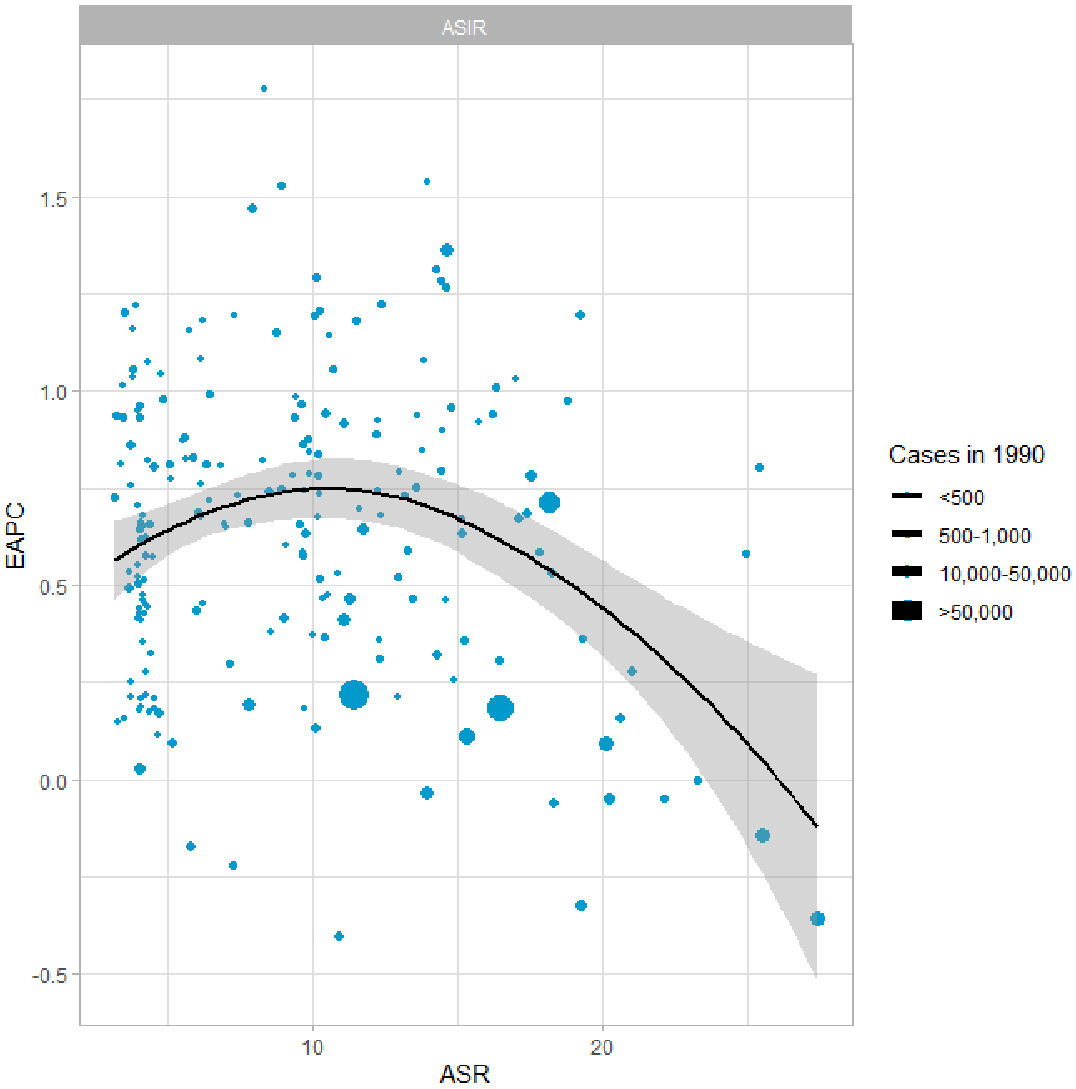


**Figure S1.** The association between EAPC and ASR analysis results: there was no significant association between EAPC and ASR. EAPC: Estimated Annual Percent Change; ASR: age-standardized incidence rate.
